# Treatment Satisfaction Among Bahraini Patients With Psoriasis: A Single Center Cross-Sectional Study Using the PsoSat Questionnaire

**DOI:** 10.7759/cureus.44354

**Published:** 2023-08-29

**Authors:** Mahmood Ali, Zainab A Toorani, Ameen Al Awadhi

**Affiliations:** 1 Dermatology, Salmaniya Medical Complex, Manama, BHR; 2 Pathology, Salmaniya Medical Complex, Manama, BHR

**Keywords:** questionnaire, biologics, satisfaction, treatment, psoriasis

## Abstract

Introduction: Psoriasis has a considerable negative impact on a patient's life. However, the treatment prescribed plays a crucial role in their quality of life. Treatment satisfaction is one of the fundamental elements when it comes to patient care for psoriasis (PsO). Dermatologists, just like any other healthcare physician, seek adherence to a specific treatment, and it has been shown that satisfaction influences treatment optimization and thereby the treatment outcome.

Methods: This was an observational cross-sectional study conducted to determine patient satisfaction with therapy and treatment of psoriasis. The final psoriasis satisfaction (PsoSat) questionnaire consisted of eight affirmations concerning treatment satisfaction. Each affirmation was rated by the patient on a 5-point Likert scale (0-4), indicating poor to perfect satisfaction levels. The study involved patients visiting the outpatient department of a multi-specialty medical complex in Bahrain, or patients contacted via teledermatology, between October 2019 and February. A total of 100 patients who were willing to participate in the survey and fulfilled the inclusion criteria, were considered.

Results: Most of the patients (89%) were moderately to very satisfied with their ongoing therapy. One patient was not satisfied with the treatment, and 10 (10%) patients reported that they were very dissatisfied with their treatment.

Conclusion: Since psoriasis can negatively impact a patient’s quality of life, treatment satisfaction is an essential factor when it comes to patient care. Dermatologists are always on the lookout for specific treatments that provide excellent results and satisfy a patient’s needs.

## Introduction

Treatment satisfaction is one of the fundamental elements when it comes to patient care for psoriasis (PsO). Dermatologists seek adherence to a specific treatment, and satisfaction especially influences treatment optimization and thereby the treatment outcome. A study showed that up to half of patients with chronic diseases compromise their treatment due to a lack of compliance with medication. Dissatisfaction, frequently not measured, was found to be the culprit [1,2].

As PsO has a considerable negative impact on patients lives, the treatment prescribed plays a crucial role in their quality of life [3]. Often, physicians take a stepwise approach to finding the most suitable treatment plan, starting with topical options such as phototherapy, then escalating to traditional systemic agents, and finally to biologicals. This process of trial and error can be frustrating from the patient’s viewpoint [4,5].

Nowadays, numerous effective therapies can achieve total control of PsO disease activity; however, it has been shown that up to 40% of patients have adherence issues in the long term. The main reasons for not being compliant with treatment are still not fully understood, but treatment dissatisfaction is thought to be a key factor. This demonstrates that satisfaction with treatment for any condition has become a crucial area for research [6,7].

Satisfaction with treatment is crucial as it measures the level to which a physician can assess the patient's health demands. It mirrors the patient's experience with their treatment journey, its duration, and its result. Various studies showed the development of treatment satisfaction instruments or measures to assess patient experiences with a certain therapeutic regimen. As much as those measures are essential for managing a certain condition, it all depends on how they are utilized in a treatment setting [8]. 

In order for a physician to aim for long-term compliance with a treatment, satisfaction measures are integral when deciding on a treatment plan. Defining ways to properly utilize those instruments is paramount to the advancement of patient care in dermatology, both in a clinical setting and for research purposes. Moderate to severe PsO accounts for around 25% of all cases, and those patients can experience a greater negative impact on their well-being and quality of life when compared to other chronic illnesses. Numerous surveys in various countries revealed that patients with moderate to severe PsO can be greatly undertreated, leaving them with a great burden of disease. Additionally, a huge number of patients were not satisfied with their ongoing treatments, leaving a great deal of dissatisfaction and ending up with frequent visits to the physician’s office [9,10].

A large population-based survey by Lebwohl et al. on patients suffering from PsO and psoriatic arthritis (PsA), emphasized that although treatment satisfaction was fairly high among patients with non-severe symptoms, many of those with severe disease were actually undertreated; for example, more than 80% of PsO patients were receiving no treatment or only topical treatment [4]. Another recent multinational large-scale survey on PsO and PsA set its sights on gathering real data on the influence of PsO and PsA treatments on the daily lives of patients. The study measured therapy, satisfaction, and the patients insight into therapy. Many physicians in North America and Europe acknowledge the unmet needs for treatment, mainly regarding the extended safety and efficacy of current therapies [11].

A national survey by Horn et al. conducted in the U.S., assessed whether patients with uncontrolled PsO are kept on systemic treatments. It concluded that around 40% were not on treatment. For those with severe disease, 26% were on systemic drugs, phototherapy alone, or a combination. Around 35% were on topical treatment only, and 39% did not receive any treatment [12].

It is not yet understood why patients with moderate to severe PsO have yet to receive adequate health care. A multinational cross-sectional study by Nast et al. looked for hurdles to the treatment of moderate to severe PsO with systemic therapy. It showed that safety worries are the main barrier to the use of systemic therapies [10]. Biological treatments, on the other hand, were not prescribed frequently due to factors relating to cost [10].

The objective of this study was to compare the satisfaction of patients with mild-to-severe PsO with the current therapies available in Bahrain using the Psoriasis Satisfaction (PsoSat) questionnaire, a new questionnaire in clinical practice that assesses satisfaction with specific treatment modalities. It identifies the level of satisfaction with the patient’s current treatment and the side effects that are bothersome. It also identifies if the current therapy is conducted too long without success and if it burdens the patient, as well as which group of psoriatic patients are most happy with their treatment.

## Materials and methods

This was an observational cross-sectional study to determine patient satisfaction with treatment of psoriasis. The PsoSat Questionnaire comprised eight statements regarding treatment satisfaction. Each statement was rated on a 5-point Likert scale (0-4), which suggests the level of agreement. The PsoSat questionnaire consisted of the eight items shown in Table 1 [13].

**Table 1:** The PsoSat questionnaire PsoSat: Psoriasis satisfaction

No.	Components in the questionnaire
1	The current treatment meets my expectations
2	I am satisfied with the current treatment
3	I wish to have a more effective treatment
4	Therapy has not been modified according to outcome
5	The side effects of the treatment bother me quite a lot
6	The current therapy has been conducted for too long without success
7	The therapy itself is quite a burden to me
8	During the treatment, I have to rely on assistance

Study participants

Patients visiting the outpatient department of a multi-specialty medical complex in Bahrain or patients contacted via teledermatology between October 2019 and February 2021 were included in the study. A total of 100 patients who were willing to participate in the survey and fulfilled the inclusion criteria were considered. The inclusion criteria were patients aged ≥ 18 years with mild-to-severe PsO capable of filling out the questionnaire or answering it remotely via a telephone call. Patients were on single or multiple treatments for PsO.

Assessment tools

The overall questionnaire in the study had sections on patient demographics, their age, gender, disease characteristics (duration, ability to work during the last 12 months, inability to work, joint pain, nail involvement, comorbidities), treatment characteristics, present and past anti-psoriatic treatments, and the most preferred treatment ever received.

Ethics

Before the initiation of the study, the approval of the Research Committee for Government Hospitals in the Kingdom of Bahrain was obtained (approval no. 81180723). All objectives of the study were explained to the participants, and their participation was optional. Informed consent was obtained from all participants, and the data was collected in an anonymized manner.

Statistical analysis

The data preparation and analysis were executed using SPSS Statistics version 26.0 (IBM Corp., Armonk, NY, USA). The data are summarized as mean ± standard deviation unless otherwise stated. The numerical data for each level of the factors were checked for normal distribution by Shapiro-Wilk's test. A Mann-Whitney U test was run to determine if there were differences in satisfaction scores between the groups. A Kruskall-Wallis test was run to determine if there were differences in satisfaction scores between more than two non-normally distributed groups. Multiple comparisons were performed to see the differences between the treatments. Significance values were adjusted using the Bonferroni correction for multiple tests. The correlation between non-normally distributed numerical variables was assessed using Spearman’s correlation.

## Results

Patient characteristics

Of the 100 eligible mild-to-severe PsO patients who were asked to participate, all were willing to participate in the study. They completed the questionnaire or answered it via a telephone-guided phone call. The majority of the participants were males (66%), and 34% were females (Table 2).

**Table 2:** Characteristics of the study participants

Category		n (%)
Gender	Male	66 (66)
	Female	34 (34)
Age in years	18 – 25	5 (5)
	25 – 35	15 (15)
	35 – 45	22 (22)
	45 – 55	29 (29)
	55 – 65	21 (21)
	>65	8 (8)
Comorbidities	Diabetes	13 (13)
	Hypertension	20 (20)
	Cardiovascular	12 (12)
	Dyslipidemia	22 (22)
Disease duration	<5 years	34 (34)
	5-10 years	40 (40)
	10-15 years	20 (20)
	> 15 years	6 (6)
Patients unable to work in the last 12 months		2 (2)
Joint pain		25 (25)
Nail involvement		18 (18)

The mean age of males was 45.5 ± 13.7 years, while the mean age of females was 46.6 ± 12.9 years. There were 20 (20%) patients with hypertension and 22 (22%) with dyslipidemia. Most of the patients (40%) had been suffering from psoriasis vulgaris for five to 10 years. Around 5% of the patients were not capable of working in the last 12 months due to their condition. Only two (2%) patients reported that they were unable to work due to their disease. There were 25% of patients with joint pain and 18% with nail involvement.

Therapy during the study

The majority of the patients (62%) were on biological therapy (adalimumab, etanercept, infliximab, and ustekinumab) (Table 3). Around 18% were exclusively on topical therapy (Daivobet ointment, mometasone, or betamethasone cream), 12% were on traditional systemic therapy, and only 8% received ultraviolet B (UVB) phototherapy.

**Table 3:** Treatment experience UVB: Ultraviolet B

Therapy	n (%)
Topical therapy (exclusively)	18 (18)
Phototherapy (UVB)	8 (8)
Traditional systemic therapy, methotrexate, cyclosporine, acitretin	12 (12)
	10 (10)
	1 (1)
	1 (1)
Biologics (adalimumab, etanercept, ustekinumab, infliximab)	62 (62)

Treatment satisfaction (PsoSat questionnaire)

Most of the patients (89%) were moderately to very satisfied with their ongoing therapy (Table 4). One patient was not satisfied with the treatment, and 10 (10%) patients reported that they were very dissatisfied with their treatment. Around 81% of patients reported that their expectations were met, and 11% reported that their expectations were not met at all (Table 5).

**Table 4:** Patient satisfaction with psoriasis treatment

Satisfaction level	n (%)
Very satisfied	81 (81)
Moderately satisfied	8 (8)
Not satisfied	1 (1)
Very dissatisfied	10 (10)
No data	0 (0)

**Table 5:** Distribution of patients according to satisfaction levels A: Perfect agreement; B: Substantial agreement; C: Moderate agreement; D: Slight agreement; E: Poor agreement

Items	A	B	C	D	E	No answer
1: The current treatment meets my expectations n (%)	64 (64)	17 (17)	6 (6)	2 (2)	11 (11)	0 (0)
2: I am satisfied with the current treatment n (%)	60 (60)	21 (21)	7 (7)	2 (2)	10 (10)	0 (0)
3: I wish to have a more effective treatment n (%)	7 (7)	13 (13)	5 (5)	20 (20)	55 (55)	0 (0)
4: Treatment is marking time n (%)	9 (9)	5 (5)	1 (1)	15 (15)	70 (70)	0 (0)
5: The side effects of the treatment bother me quite a lot n (%)	8 (8)	0 (0)	6 (6)	7 (7)	79 (79)	0 (0)
6: The current therapy is conducted for too long without success n (%)	9 (9)	2 (2)	1 (1)	3 (3)	85 (85)	0 (0)
7: The therapy itself is quite a burden to me n %)	9 (9)	1 (1)	2 (2)	10 (10)	78 (78)	0 (0)
8: During the treatment I have to rely on assistance n (%)	5 (5)	3 (3)	3 (3)	7 (7)	82 (82)	0 (0)

Around 25% of patients reported that they wished to have more effective treatments, and 75% of patients did not wish to switch to another treatment. Eighty-five patients (85%) reported that their treatment does not take much of their time, and only fourteen patients (14%) reported that they experienced some sort of side effect during their therapy. Eighty-eight patients (88%) reported that their therapy was of no burden to them whatsoever; two (2%) reported moderate agreement; and only 10 patients (10%) reported otherwise. Eighty-nine patients (89%) relied on no assistance whatsoever during their therapy, while only eight patients (8%) felt that they had to rely on assistance during treatment.

Association of current treatment with satisfaction level

**Figure 1:**
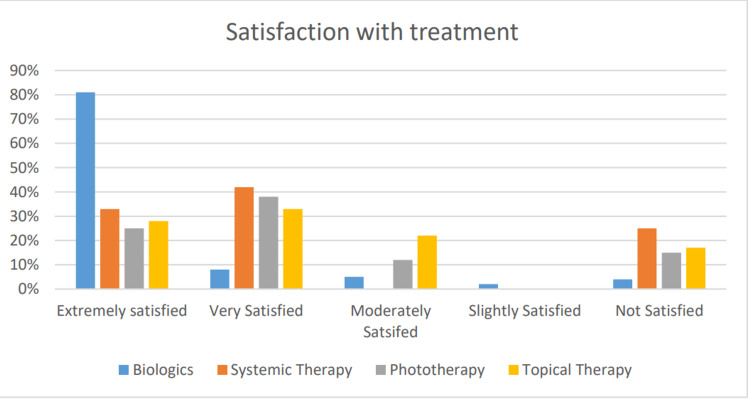
Column chart showing the percentage of patients as per varied treatment satisfaction levels

Out of all the participants currently receiving biologicals, 81% expressed extreme treatment satisfaction, compared to 33% who were on conventional systemic therapy, 25% on phototherapy, and 27% on topical therapy. A score of 4 or 3 on the Likert scale was provided by 12% of patients treated with biologics, 41% of patients on systemic therapy, 50% of patients on phototherapy, and 55% of patients on topical therapy (Figure 1).

**Figure 2:**
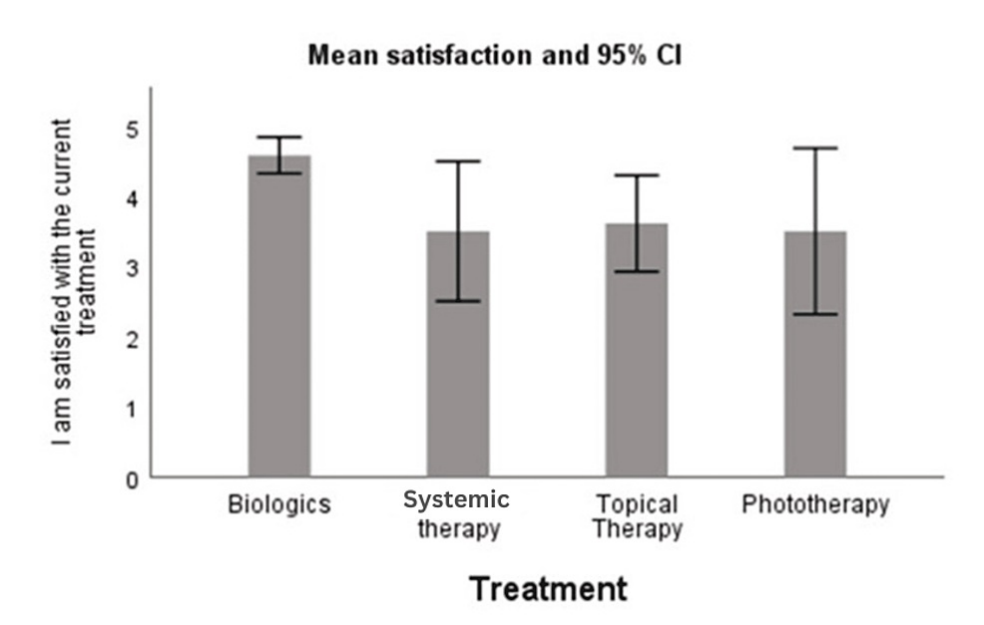
Column chart showing mean satisfaction levels along with 95% CI as per varying treatments

Only 6% of the patients on biologics reported that they were not satisfied with their treatment, compared to 25% on systemic therapy, 25% on phototherapy, and 16% on topical therapy. The satisfaction assessed on a 5-point Likert scale was not normally distributed for all treatments, as assessed by Shapiro-Wilk's test (p < 0.05). A Kruskall-Wallis test was run to determine if there were differences in the satisfaction score between the four treatments. The median scores for all the treatments were statistically significantly different (p < 0.0001). The results of pairwise comparisons showed that the biological treatment had a higher satisfaction level than all the other treatments (p < 0.01). There was no significant difference between the other three treatments (p > 0.05) (Figure 2).

Effect of disease duration on treatment satisfaction

**Figure 3:**
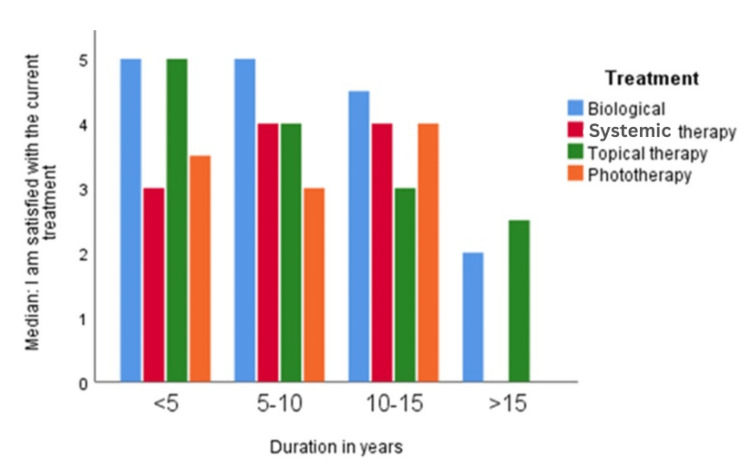
Column chart showing the mean satisfaction of current treatments according to duration

The satisfaction assessed on a 5-point Likert scale was non-normally distributed for all treatment durations, as assessed by Shapiro-Wilk's test (p < 0.05). A Kruskall-Wallis test was run to determine if there were differences in the satisfaction score between the four treatments and the duration of the disease. Median scores for the treatment and the disease durations were statistically significantly different (p < 0.0001). The results of pairwise comparisons showed that for the duration of treatment between zero and five years and five to 10 years, the satisfaction level was higher compared to the groups with treatment durations of 10 to 15 years and more than 15 years (p < 0.01) (Figure 3).

There was no significant difference between the durations of zero to five, five to 10 years, 10 to 15 years, and more than 15 years of treatment duration (p > 0.05).

Differences between the genders with regard to all items of the PsoSat

A Mann-Whitney U test was run to determine if there were differences in the satisfaction score between males and females. The distributions of the satisfaction scores for males and females were similar, as assessed by the visual inspection. The median satisfaction scores for males and females were not statistically significantly different (p = 0.974).

Correlation of age with treatment satisfaction

There was a weak negative correlation between age and the satisfaction score (p = -0.207), which was statistically significant (p = 0.039). This indicated that as age increases, the treatment satisfaction score decreases.

## Discussion

Treatment satisfaction is an extremely important topic, as several studies have noted that many patients need early intervention when treatment is not up to expectations. It was postulated that PsO nowadays is not just a disease of the skin but a systemic disease, which necessitates early treatment strategies in order to provide early control of the disease [14]. The sample of patients who participated in this study comprises primarily Bahraini psoriatic patients undergoing treatment using various treatment modalities while varying greatly in age, comorbidities, and treatment strategies. In the present study, the majority of patients were male (66%), reflecting a higher prevalence of Bahraini male patients with psoriatic disease. We believe that the sample of participants is representative of the population. In our assessment, the greatest satisfaction level was perceived by patients on biologicals, followed by those on traditional systemic medications, topical medications, and lastly, phototherapy. Our study proposed that older patients in general preferred topical therapy. It could be due to the fact that older participants are hesitant to use conventional therapy as they are more likely to have comorbidities and are on other medications that may interact with anti-psoriatic medications.

A study by Geredes et al. showed that patients with severe PsO are receiving significantly more different systemic drugs on average than the general population, with the most prominent difference in multidrug treatment [12]. The proportion of patients in our study receiving biologics was high (62%), followed by topical therapy (18%) and systemic therapy (12%). In a study by Armstrong et al., 5604 patients with PsO or PsA completed the survey, and the results showed that around 35% to 50% of patients with mild PsO were untreated, followed by 23.6% to 35.5% of those with moderate disease and 9.45% to 29.7% of those with severe disease [13]. Of those patients, 29.5% with moderate disease and 21.5% with severe disease were on topical treatment only [12]. The study concluded that 52.3% of participants with PsO and 45.5% of them with PsA were unhappy with their therapy [12]. This finding drastically differs from our study, as the majority of Bahraini patients were satisfied with their therapy, with most of their expectations being met. Nearly 81% of the participants in our study were fully satisfied with their treatment plan. However, 24% of patients expressed their wish for a more effective treatment, and only 12% reported that their treatment continued for too long without progress.

A study by Finlay et al. showed that biological therapy offers a glimpse of hope for psoriatic patients not well controlled by other agents, which leads to drastic improvement in their quality of life and greater satisfaction with their therapy [14]. This was in line with our findings, as patients were extremely happy with their treatment. Treatment satisfaction is a separate patient-reported result, mainly focusing on the patient’s evaluation of a specific drug and the outcomes associated with the drug. Shikiar et al. reported that satisfaction with medication is narrowly focused and should be distinguished from other aspects of satisfaction [15]. Medication satisfaction is a type of patient-reported outcome but is distinguished from other patient-reported outcomes, specifically health-related quality of life (HRQL) and self-reported symptoms [1].

Our data suggest that patients taking ustekinumab report the highest levels of satisfaction, followed by adalimumab, infliximab, and etanercept. In a study by Lucka et al., ustekinumab and infliximab were found to be superior to adalimumab and etanercept. Infliximab had the fastest onset of action, followed by ustekinumab and adalimumab [16,17]. In a study by van den Reek et al., ustekinumab showed a better overall drug survival than etanercept and a trend towards a better overall drug survival than adalimumab [18]. After one year, patients reported being 'happy' in 79% of episodes and 'unhappy' in 21% [19]. The observation was in line with our study's findings. Treatment satisfaction for patients on systemic therapy was high, with nearly 75% of patients reporting being extremely satisfied or very satisfied. Most of the patients were on methotrexate, with one on cyclosporine and one on acitretin. This was almost similar to the two studies by Kavanaugh et al. and Lebwohl et al., where levels of dissatisfaction with systemic treatments were high, with 30.5% of respondents indicating dissatisfaction with methotrexate, mainly due to its side-effects [17,20].

Nowadays, the main emphasis when it comes to the measurement of patient satisfaction is the use of the PsO area and severity index, which disregard the patient's true emotions. Lehwoul et al. evaluated how the severity of the disease is determined differently by dermatologists and patients; patients with PsO consistently reported itching, which is usually not present in the assessment tools and was found to be a crucial factor in assessing the disease severity [17]. Physicians, however, reported the size or location of lesions as important factors contributing to the overall disease severity [3].

It has been shown that if patients are satisfied with their treatment plan, they tend to be more adherent to it, which leads to optimal results. It is a crucial area of research to optimize patients treatment, reflect patient-specific care, and assimilate patients viewpoints into real-world clinical practice. In a study by Atkinson et al., it was shown that the Treatment Satisfaction Questionnaire for Medication (TSQM) is a valid measurement of patients satisfaction. Data suggests that it is also a good predictor of patients medication adherence across different types of medication and patient populations [19].

The major limitations of our study are that it is a single-center study and that the sample size was limited. Many patients also had a long disease duration, and some had a refractory course. More than half were on biologics, and almost 12% of the participants used systemic medications. Evidently, satisfaction with topical treatment was expected to be higher than that of phototherapy, as the patients had milder and less refractory PsO. A reason for differences in the satisfaction level between our study and others is that biologics can be prescribed as first-line treatment in Bahrain and modified based on the patient’s response with ease.

The major advantages of this study are its examination of satisfaction in a real clinical setting with proven scoring and its assessment of preferences for specific medications. The highest satisfaction level was observed with biologicals, where ustekinumab and adalimumab were superior for skin-limited disease.

## Conclusions

The results of this questionnaire from Bahrain strongly support the findings from several other studies and countries that patients with PsO are often satisfied with their treatment if biologics are the main modality of treatment. Satisfaction with the treatment is extremely vital to PsO management. Displeasure often results in non-compliance with treatment, which may be misjudged as treatment failure. This may cause the discontinuation of a therapy that may have been effective. Currently, no proven, consistent treatment satisfaction measure is available to properly assess a specific treatment. These assessments recognize therapy efficacy, convenience, modification if necessary, and patients at risk for noncompliance. This will lead to the participation of patients in their treatment plan, which will lead to proper delivery of health care and enhanced treatment success.
